# MicroRNAs and their targeted genes associated with phase changes of stem explants during tissue culture of tea plant

**DOI:** 10.1038/s41598-019-56686-3

**Published:** 2019-12-27

**Authors:** Ying Gao, Da Li, Lu-Lu Zhang, Devajit Borthakur, Qing-Sheng Li, Jian-Hui Ye, Xin-Qiang Zheng, Jian-Liang Lu

**Affiliations:** 1 Zhejiang University Tea Research Institute, Hangzhou, 310058 P.R. China; 2 The World Vegetable Centre, Guwahati, Assam India

**Keywords:** Plant regeneration, miRNAs

## Abstract

Elucidation of the molecular mechanism related to the dedifferentiation and redifferentiation during tissue culture will be useful for optimizing regeneration system of tea plant. In this study, an integrated sRNAome and transcriptome analyses were carried out during phase changes of the stem explant culture. Among 198 miRNAs and 8001 predicted target genes, 178 differentially expressed miRNAs and 4264 potential targets were screened out from explants, primary calli, as well as regenerated roots and shoots. According to KEGG analysis of the potential targets, pathway of “aminoacyl-tRNA biosynthesis”, “proteasome” and “glutathione metabolism” was of great significance during the dedifferentiation, and pathway of “porphyrin and chlorophyll metabolism”, “mRNA surveillance pathway”, “nucleotide excision repair” was indispensable for redifferentiation of the calli. Expression pattern of 12 miRNAs, including *csn-micR390e, csn-miR156b-5p*, *csn-miR157d-5p*, *csn-miR156, csn-miR166a-3p*, *csn-miR166e*, *csn-miR167d*, *csn-miR393c-3p*, *csn-miR394, csn-miR396a-3p*, *csn-miR396* and *csn-miR396e-3p*, was validated by qRT-PCR among 57 differentially expressed phase-specific miRNAs. Validation also confirmed that regulatory module of *csn-miR167d*/*ERF3*, *csn-miR156*/*SPB1*, *csn-miR166a-3p*/*ATHB15*, *csn-miR396*/*AIP15A*, *csn-miR157d-5p*/*GST* and *csn-miR393c-3p*/*ATG18b* might play important roles in regulating the phase changes during tissue culture of stem explants.

## Introduction

In plants, small non-coding RNAs (sRNAs) range in length from 20 to 26 nt, including microRNAs (miRNAs) and small interfering RNAs (siRNAs). miRNAs with length of 20–22 nt, being produced by DICER-LIKE1, can negatively regulate gene expression at the post-transcriptional level through degradation or translational repression of the targeted mRNAs^1–3^. A large number of studies revealed that miRNAs represent as key regulators in plant developmental and physiological processes including organ morphogenesis, hormone signaling, defense response and nutrient metabolism^2,4–11^.

According to an increasing number of published reports, miRNA-mediated regulation usually plays a critical role in the development of embryos, roots and shoots *via* the regulating transcription factors and hormone-related genes. During the stage of embryogenesis, *miR393* was found to control the F-box family genes encoding TIR1 and AFB2 auxin receptors during the embryogenic transition of somatic cells in *Arabidopsis*^12^. Overexpression of *miR167* resulted in low level of *auxin response factor 6* (*ARF6*) and *ARF8* transcripts and inhibited somatic embryo formation in *Arabidopsis*^13^. In zygotic embryogenesis of *Arabidopsis*, *miR165* and *miR166* could control the transcript abundance of the *PHABULOSA* and *PHAVOLUTA* (*PHB* and *PHV*) genes which were the positive regulators of *LEAFY COTYLEDON2* (*LEC2*), while *miR160* could negatively target the *AUXIN RESPONSE FACTORS* including *ARF10*, *ARF16* and *ARF17*^9^. In cotton, a *GhmiR157a-GhSPL10* regulatory module was proved to be associated with initial cellular dedifferentiation and callus proliferation *via* hormonal and flavonoid pathways^14^. *miR166* could regulate the expression of the several class-III HD-ZIP genes and play an important role in the lateral root development^15^; and *miR396* could modulate the transition of root stem cells into transit-amplifying cells through interacting with *GROWTH-REGULATING FACTORs* (*GRFs*) in *Arabidopsis thaliana*^16^. It was proved that *miR166* and *miR156* could control the shoot apical meristem (SAM) formation^17–19^; and that *miR156* and *miR160* could modulate the shoot regeneration^20,21^, while *miR171* could influence the shoot branching^22^. In light of these evidences, it would be important to identify the miRNAs present in tea plants.

As one of the most important cash crops worldwide, tea plant (*Camellia sinensis* (L.) O. Kuntzes) is famous for its leaves production which is non-alcoholic and good for human health. Unfortunately, it is difficult for many researchers to study on the development mechanism of tea plant because of lacking a perfect plant regeneration system. Nowadays, although increasing attention is being paid on tea plantlets regeneration through organogenesis and somatic embryogenesis, a significant difference in regeneration frequency was observed from various explants, and very low frequency was usually witnessed during induction of many explants^23–28^. Therefore, investigation on mechanism of the dedifferentiation and redifferentiation during tissue culture might be useful for optimizing efficient regeneration system of tea plant.

Numerous conserved miRNAs and their targets have recently been identified in *C. sinensis*. Some of these miRNAs are responsible for cold stress^29^, *Ectropis oblique* feeding^30^ and drought stress^31^. Using small RNA (sRNA) sequencing tech, Sun and colleagues identified 69 conserved and 47 novel miRNAs related to catechins biosynthesis from the EGCG-enriched tea plant line No. 1005^32^. It was found that 175 conserved and 83 novel miRNAs were mainly present in one bud and two tender leaves of the tea plant^33^. Although there is a large amount of information on miRNA expression related to dedifferentiation and redifferentiation in some other plants, relatively little information is available for tea plant. In the present study, Illumina HiSeq 2500 technology was occupied to sequence the putative miRNAs and mRNAs for investigating their expression profiles during the dedifferentiation and redifferentiation of tea plant tissue culture. The regulatory effect of miRNAs on the targeted genes during the phase transition of tissue culture was confirmed by qPCR.

## Results

### Transcriptome sequencing and assembly

Average of the obtained clean data for each sample exceeded 6GB after sequencing. A total of 194,014 transcripts was achieved from the stem explants, primary calli, redifferentiated roots and shoots through de novo assembly. 100,099 unigenes were obtained from the transcription data of these samples, with 789 bp in average length and 1,514 bp in N50 length; and 22,540 unigenes had a length of above 1000 bp, accounting for 22.52% of the total sequence number (Table 1).

**Table 1:** Summary results of the unigene assembly*.

Length Range	Transcript	Unigene
200–300 bp	44,334 (22.85)	37,595 (37.56)
300–500 bp	34,370 (17.72)	22,368 (22.35)
500–1000 bp	40,707 (20.98)	17,596 (17.58)
1000–2000 bp	42,240 (21.77)	12,719 (12.71)
2000 + bp	32,363 (16.68)	9,821 (9.81)
Total Number	194,014	100,099
Total Length (bp)	214,331,245	78,961,581
N50 Length (bp)	1,881	1,514
Mean Length (bp)	1104.72	788.83

*Data in parentheses represent the percentage (%).

### High-throughput sequencing of sRNAs

After sRNA sequencing, more than 21 M raw reads and 15 M clean reads were obtained from each biological sample, around 1/10 of clean reads could mapped onto the reference transcriptome unigenes, and the mapped reads were 2.76 M and 2.62 M, 2.13 M and 1.15 M in stem explants, primary-calli, regenerated roots and shoots, respectively (Table 2). Comparison showed that a certain proportion of rRNA and tRNA, as well as a small amount of snoRNAs and Repbase repeat sequences were contaminated in clean reads, but sRNA proportion exceeded 75% except in regenerated shoots (Table 3).

**Table 2:** Average data of sRNA obtained from different tissue culture samples.

Samples	Raw reads	Low quality reads	Containing ‘N’reads	Length <18 nt	Length >30 nt	Q30 (%)	Clean reads	Mapped reads
S_Explant	25,090,444	0	0	1,133,182	509,916	98.68	23,447,346	2,760,666
S_Primary callus	24,393,083	0	0	4,219,353	624,187	98.66	19,549,543	2,617,031
S_Root	24,294,099	0	0	832,884	811,259	98.74	22,649,956	2,125,327
S_Shoot	21,696,247	0	28	4,541,061	1,494,147	98.48	15,661,011	1,145,331

**Table 3:** Sequence count of the small RNAs annotated in different database*.

Type	S_Explant	S_Primary callus	S_Root	S_Shoot
Total	23,447,346 (100.00)	19,549,543 (100.00)	22,649,956 (100.00)	15,661,011 (100.00)
rRNA	3,431,224 (14.63)	4,345,193 (22.23)	3,153,731 (13.92)	5,518,651 (35.24)
scRNA	0 (0.00)	0 (0.00)	0 (0.00)	0 (0.00)
snRNA	1 (0.00)	1 (0.00)	1 (0.00)	1 (0.00)
snoRNA	2,503 (0.01)	5,610 (0.03)	4,315 (0.02)	4,316 (0.03)
tRNA	77,728 (0.33)	264,152 (1.35)	213,497 (0.94)	826,990 (5.28)
Repbase	10,500 (0.04)	13,524 (0.07)	11,109 (0.05)	10,059 (0.06)
sRNA	19,925,390 (84.99)	14,921,063 (76.32)	19,267,303 (85.07)	9,300,994 (59.39)

^*^Data in parentheses represent the percentage (%).

**Figure 1:**
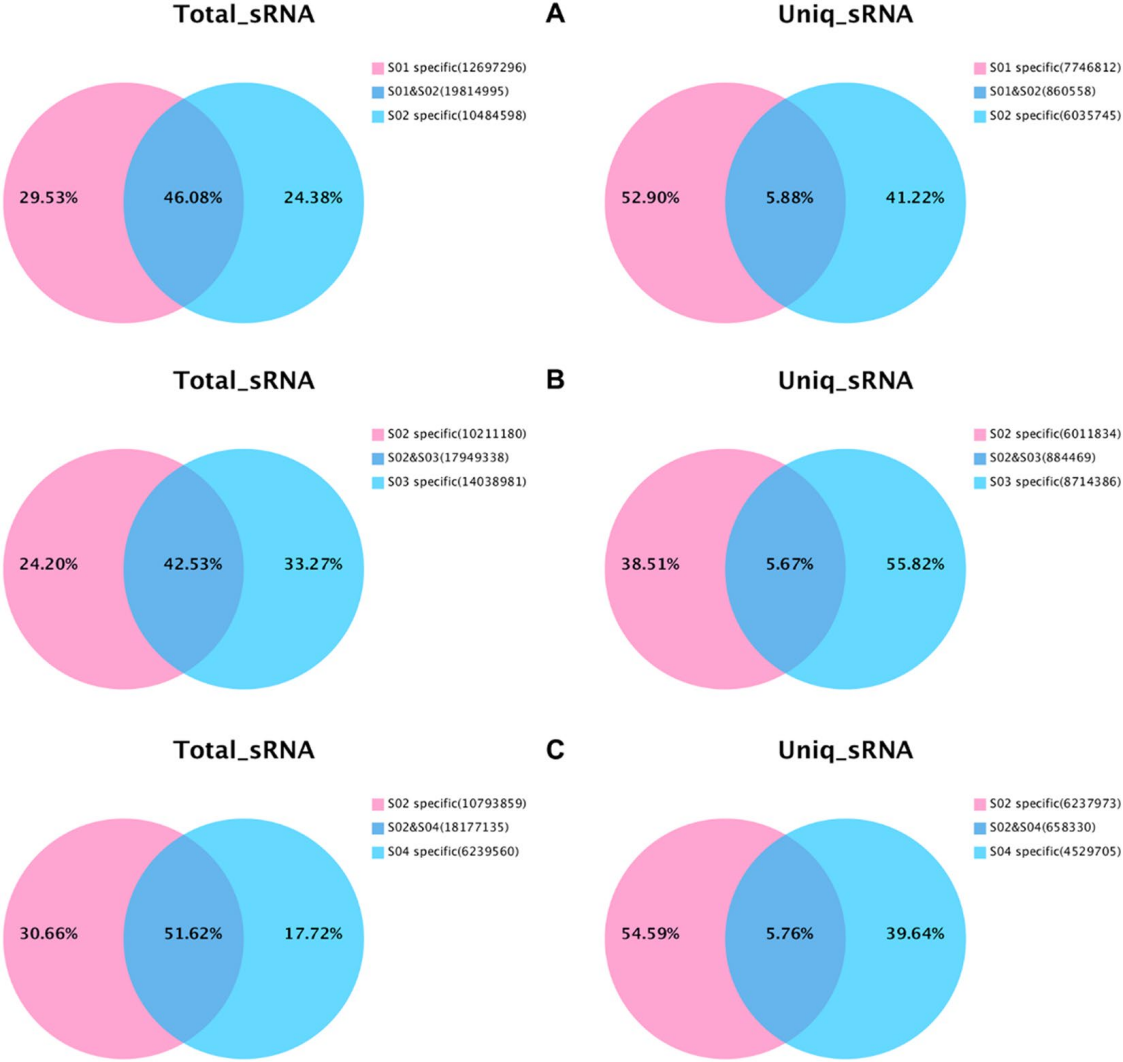
Venn diagram of sRNA common and unique sequence number among various samples. The types of unique sequences (represented by Unique reads) and the number of common sequences (represented by Total reads) between different samples were counted. The sRNA sequence types were counted with the reads after removal of redundancy; the number of sRNA sequences was counted by all reads. (**A**) S_Explant (S01) *vs* S_Primary callus (S02); (**B**) S_Primary callus (S02) *vs* S_Root (S03); (**C**) S_Primary callus (S02) *vs* S_Shoot (S04).

Quantity proportion of the common reads and type proportion of the unique reads in the sRNA sequences were evaluated among the stem explant and its derivatives. Only a few unique reads (5.66–5.88%) shared their common types among these four samples, however, these common types represented 38.51–55.82% of the total reads (Fig. 1), indicating that the sRNAs varied significantly during dedifferentiation and redifferentiation, and a few proportion of the sRNAs with abundant expression might play important roles during phase transition.

### miRNAs and their expression patterns during phase change

After blasting with the miRNA database miRBase (v21), a total of 59 conserved plant miRNAs were identified, in which 56, 54, 50 and 50 ones were observed in the explant, primary callus, regenerated root and shoot, respectively (Table 4). A total of 139 potential novel miRNAs were also predicted by the software miRDeep2^34^ on Bayesian model, based on the distribution information of the reads on the precursor sequence and precursor structure energy information (RNAfold randfold), in which 133, 117, 126 and 113 ones were observed in these four types of biological samples, respectively. Thus, 198 miRNAs were identified in this study (Supplementary Table S1). These miRNAs belonged to 53 families, such as *miR166*, *miR396*, *miR159*, *miR535*, *miR160*, *miR482*, *miR171_1* and so on.

**Table 4:** Count result of the miRNAs and targeted mRNAs*.

Samples	All miRNA	Conserved miRNAs	Novel miRNAs	Targeted genes
S_Explant	189	56	133	7949
S_Primary callus	171	54	117	6825
S_Root	176	50	126	7230
S_Shoot	163	50	113	6812
Total	198	59	139	8001

*There were 2 novel miRNAs without predicted target genes in 198 miRNAs.

**Figure 2:**
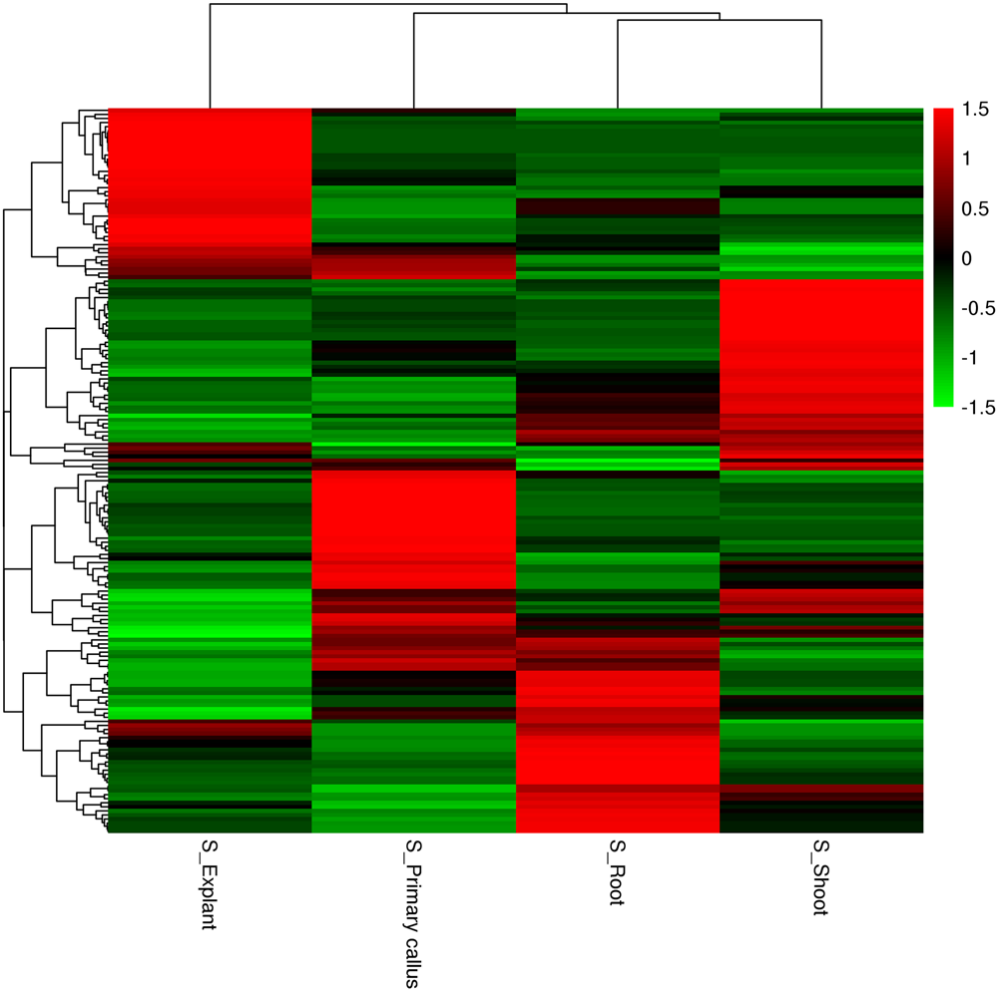
The heat-map of 178 different expressed miRNA shared in the 4 samples, based on Z-score normalized TPM values in eight internode segments.

According to calculation of TPM values of the miRNAs and the threshold of *q* value < 0.005 & |log2(Fold change) > 1| between different samples, 178 differentially expressed miRNAs, including 52 conserved and 126 novel miRNAs, were screened out from the detected 198 miRNAs. 94 miRNAs (24 conserved and 70 novel) were up-regulated during dedifferentiation of the stem explant. Among them, 25 miRNAs (6 conserved and 19 novel) were up-regulated furthermore while 69 miRNAs (18 conserved and 51 novel) were down-regulated during root redifferentiation; similarly, 36 miRNAs (8 conserved and 28 novel) were up-regulated while 58 miRNAs (16 conserved and 42 novel) were down-regulated during shoot redifferentiation. Meanwhile, 84 miRNAs (28 conserved and 56 novel) were down-regulated during phase change from stem explant to primary callus, of which 58 (20 conserved, 38 novel) and 52 (18 conserved, 34 novel) ones were up-regulated during regeneration of root and shoot, while 26 (8 conserved, 18 novel) and 32 (10 conserved, 22 novel) ones were down-regulated during these regenerations. Cluster analysis visually revealed that expression levels of these miRNAs were significantly different among explant, primary callus, regenerated root and shoot, especially between explant and its derivatives (Fig. 2), indicating that these differentially expressed miRNAs might be involved in regulation of phase change through its effect called posttranscriptional gene silencing.

### Potential target genes of the miRNAs

196 miRNAs (59 conserved and 137 novel) were predicted to be able to target 8001 potential function genes. As for differentially expressed 178 miRNAs, 1811 genes were potentially targeted by 52 conserved miRNAs, while 6025 genes were done by 126 novel miRNAs. The targeted genes were blasted with COG, GO, KEGG, KOG, Pfam, SwissProt, eggNOG, and NR databases. A total of 4264 targets were annotated, including 2964 sequences with a length greater than 1000 bp and 1300 sequences with a length between 330 bp and 1000 bp (Table 5). Most of these targets were mainly related to the function of “transcription” (such as transcription factors and growth-regulating factors), “signal transduction mechanisms” (such as receptor protein kinase) and “posttranslational modification, protein turnover, chaperones” (Supplementary Table S2), and significantly enriched in the GO items of “growth” and “signaling” in the biological process, and “nucleoid” and “cell junction” in cellular component, as well as “electron carrier activity” and “enzyme regulator activity” in the molecular function (Supplementary Fig. S1).

**Table 5:** Annotation count of the genes targeted by miRNAs.

Database	Annotated number	300 <= length < 1000 (bp)	length >= 1000 (bp)
COG	1345	234	1111
GO	2417	669	1748
KEGG	1518	391	1127
KOG	2485	656	1829
Pfam	3203	674	2529
Swissprot	2844	716	2128
eggNOG	4040	1159	2881
Nr	4161	1219	2942
All	4264	1300	2964

**Figure 3:**
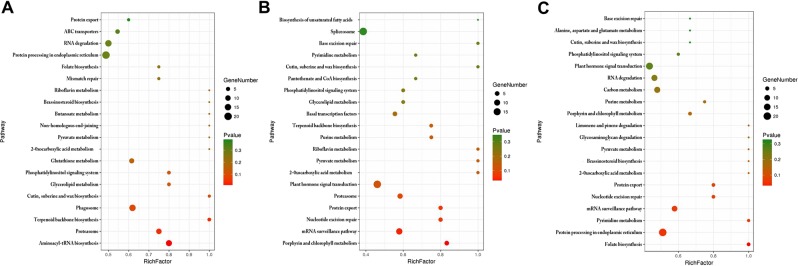
KEGG analysis of the targeted genes during phase change of tissue culture. (**A**) S_Explant vs S_Primary callus; (**B**) S_Root vs S_Primary callus; (**C**) S_Primary callus vs S_Shoot.

A total of 1518 targets were annotated in various pathways after being blasted with KEGG database (https://www.kegg.jp/kegg/kegg1.html)^35^. Among them, 710 targets were observed during the dedifferentiation stage and enriched in KEGG pathway of “aminoacyl-tRNA biosynthesis”, “proteasome”, “terpenoid backbone biosynthesis”, “phagosome”, “cutin, suberine and wax biosynthesis”, “glycerolipid metabolism”, “phosphatidylinositol signaling system” and “glutathione metabolism” (Fig. 3A). During the roots regeneration, 613 targets were mainly enriched in the KEGG pathway of “porphyrin and chlorophyll metabolism”, “mRNA surveillance pathway”, “nucleotide excision repair”, “protein export”, “proteasome”, “plant hormone signal transduction”, “2-oxocarboxylic acid metabolism”, “pyruvate metabolism” and “riboflavin metabolism” (Fig. 3B); during shoots regeneration, 624 targeted genes were mostly enriched in pathway of “folate biosynthesis”, “protein processing in endoplasmic reticulum”, “pyrimidine metabolism”, “mRNA surveillance pathway”, “nucleotide excision repair”, “protein export”, “2-oxocarboxylic acid metabolism”, “brassinosteroid biosynthesis”, “pyruvate metabolism” and “porphyrin and chlorophyll metabolism” (Fig. 3C). Obviously, genes related to the pathway of “aminoacyl-tRNA biosynthesis”, “proteasome” and “glutathione metabolism” might be necessary for cell division and replication during dedifferentiation, which play an important role in the stress and defense of phase change^36,37^; meanwhile, genes associated with pathway of “porphyrin and chlorophyll metabolism”, “mRNA surveillance pathway”, “nucleotide excision repair”, “protein export”, “2-oxocarboxylic acid metabolism” and “pyruvate metabolism” might play important roles in plastid rebuilt^37^, cellular stress^38^, DNA repair^39^ and secondary metabolism during the redifferentiation.

### Expression validation of phase-specific miRNAs and potential targets

**Figure 4:**
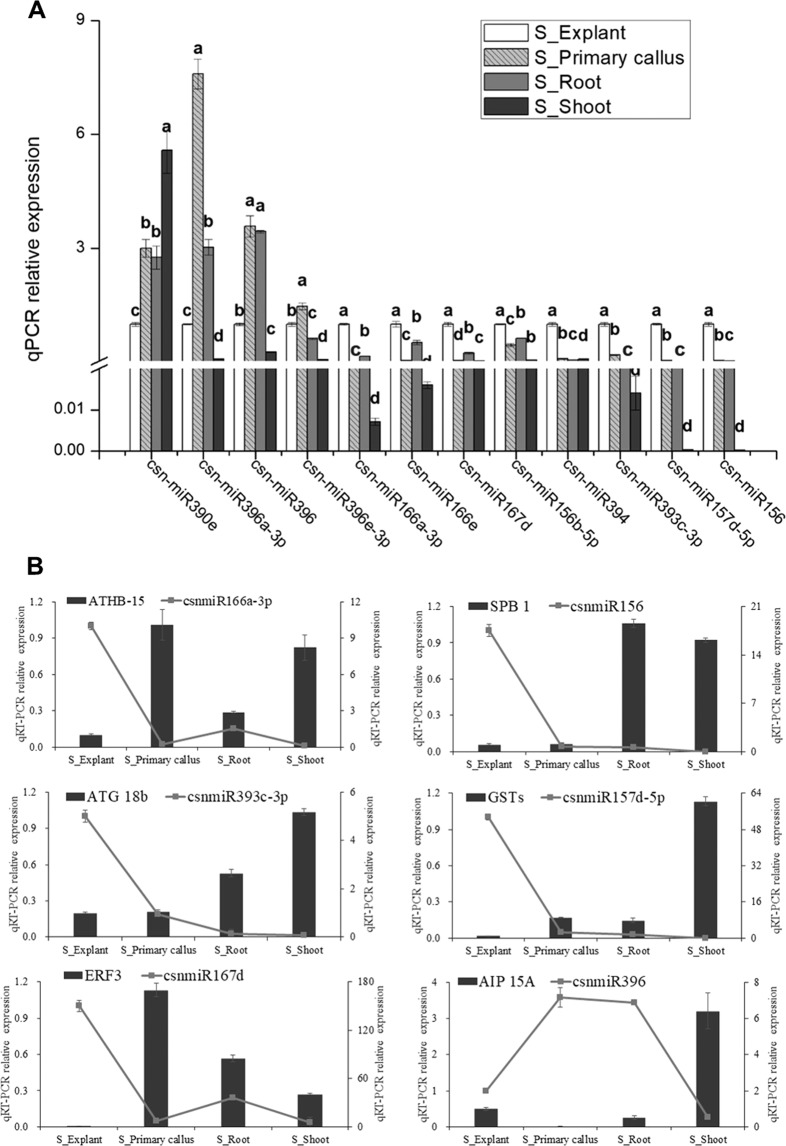
(**A**) expression validation of phase-specific miRNA by qPCR; (**B**) expression relationship between 6 phase-specific miRNAs and their targeted genes, the line represented the expression level of miRNAs and corresponded to the principal ordinate axis (left Y-axis), the histogram represented expression level of the targeted genes and corresponded to the secondary ordinate axis (right Y-axis). Different letters indicated significant differences at p < 0.05 (lower letters for miRNA expression and uppercase for targeted gene expression).

Among the 178 differentially expressed miRNAs, 57 miRNAs (28 conserved and 29 novel) exhibited obvious phase-specific expression pattern, *i.e*., extremely up- or down-regulated expression at one status among explants and the relevant derivatives (Supplementary Fig. S2). Expression pattern of 12 miRNAs among them was validated by qPCR (Fig. 4A), in phase-specific view, low expression of *csn-miR390e* in stem explants, down-regulated expression of *csn-miR156b-5p*, *csn-miR157d-5p* and *csn-miR156* in regenerated shoots; whilst high expression of the *csn-miR166a-3p*, *csn-miR166e*, *csn-miR167d*, *csn-miR393c-3p* and *csn-miR394* in stem explants, up-regulated expression of *csn-miR396a-3p*, *csn-miR396* and *csn-miR396e-3p* in primary-callus.

To validate the effect of the phase-specific miRNAs on the corresponding function mRNAs during phase change of tissue culture, combined expression analysis of the miRNA and the targeted gene was carried out by qPCR. It was found that modulation of the *csn-miR167d*/*ERF3* (*Ethylene-responsive transcription factor ERF003*), *csn-miR156*/*SPB1* (*Squamosa promoter-binding protein 1*), *csn-miR166a-3p*/*ATHB15* (*Homeobox-leucine zipper protein ATHB 15*), *csn-miR396*/*AIP15A* (*Auxin induced protein 15A*), *csn-miR157d-5p*/*GST* (*Glutathione S-transferase*) and *csn-miR393c-3p*/*ATG18b* (*Autophagy-related protein 18b*) might play important roles during phase changes because the expression pattern of the miRNAs was negatively correlated with the corresponding targets (Fig. 4B).

## Discussion

**Figure 5:**
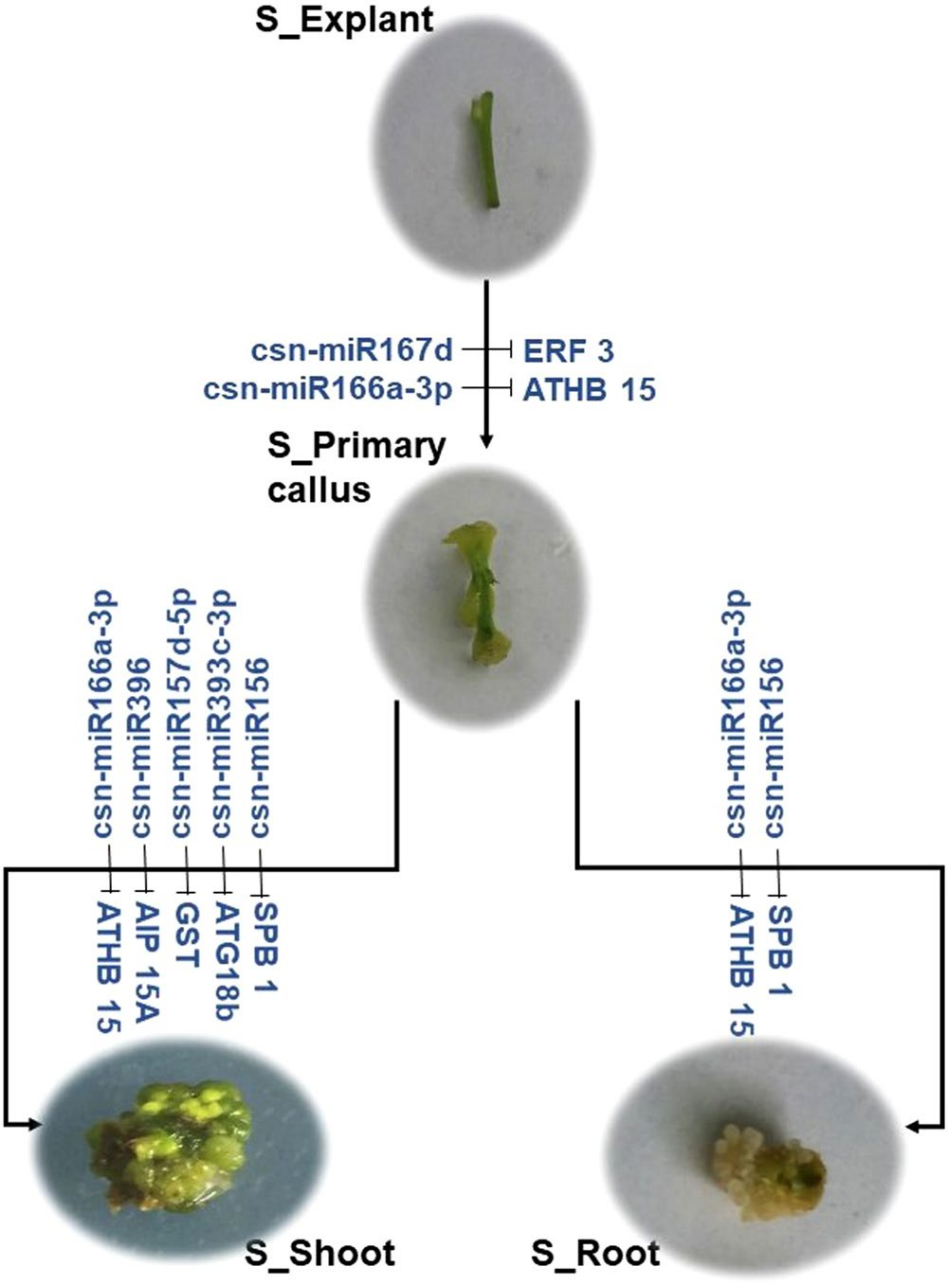
Potential regulatory module of phase-specific miRNA and the targeted genes during phase change of the tissue culture of stem explants.

MicroRNAs have been considered as significant regulators in embryogenesis, roots and shoots development through modulating the expression of targeted genes. In this paper, relationship between miRNAs and mRNAs was investigated during dedifferentiation and redifferentiation of tea explant through sRNAs and mRNAs sequencing. Interestingly, modulation of *csn-miR167d*/*ERF3*, *csn-miR156*/*SPB1*, *csn-miR166a-3p*/*ATHB15*, *csn-miR396*/*AIP15A*, *csn-miR157d-5p*/*GST* and *csn-miR393c-3p*/*ATG18b* might closely relate to dedifferentiation and redifferentiation during tissue culture of stem explants (Fig. 5).

During primary callus formation, significantly low expression of *csnmiR167d* was observed, while expression of its target *ERF003* was specifically up-regulated. *ERF003*, one of the ethylene-responsive transcription factors, acts as a transcriptional activator through binding to the GCC-box promoter element and regulates expression of the genes involved in the response to stress factors and components of stress signal transduction pathways^40^. According to our study, *miR167d* might be an important regulator of the signaling pathway through modulating the *ERF003* during callus formation of stem explant. In *Arabidopsis*, over-expression of *miR167* would inhibit the somatic embryos formation *via* negatively regulating *ARF6* and *ARF8*^13^, which implied that low expression of *miR167* might be in favor of plant embryonic callus formation. Many previous studies revealed that ethylene could induce callus formation and plant regeneration in citrus^41^, apple^42^, barley^43^ and *Arabidopsis*^44^; and that up-regulated ethylene biosynthesis would promote the process of embryogenesis through activating the ethylene-responsive transcription factor in soybean^45^. Thus, *miR167d*/*ERF003* module might play an important influence in dedifferentiation of stem explant.

As well known, the highly conserved *miR156/SPL (Squamosa promoter- binding-like protein)* module was reported to regulate stress response^46^, leaf and fruit development^47,48^, grain size^49,50^, shoot regeneration^21^, and callus induction^51^. In *Arabidopsis*, *miR156* could respond to auxin signaling and modulate the target *SPL* 3/9/10, consequently control the quantity of lateral roots^52^. In present study, low expression of *miR156* and up-regulated expression of *SBP1* were observed during root and shoot regenerations. Transcriptional factor SBP1, an ortholog of SPL, can bind to the AP1 promoter and regulate the expression of *SQUAMOSA* involved in development^53^. Thus, the effect of *miR156-SPB1* module on the redifferentiation is worthy to be further studied.

Low expression of the *miR396, miR166a-3p, miR157d-5p and miR393c-3p*, coupled with up-regulated expression of *AIP15A*, *ATHB15*, *GST* and *ATG18b* were observed during shoot regeneration, indicating module of *miR396*/*AIP15A, miR166a-3p*/*ATHB15, miR157d-5p/GST and miR393c-3p*/*ATG18b* might be responsible for shoot redifferentiation. In *Cymbidium*, promoted auxin biosynthesis was required for efficient shoot regeneration^54^. *AIP15A* belongs to the early auxin-responsive SAUR family gene. Up-regulated expression of this gene suggested that auxin and auxin signaling pathway were both essential for shoot regeneration of tea plant^55,56^. Previous research also showed that *ATHB15* can exert its regulation at the early stage of shoot induction^57^. *GST* is involved in the conjugation of reduced glutathione to a wide number of exogenous and endogenous hydrophobic electrophiles and plays a detoxification role under stress conditions including pathogens infection, cold, drought and wounding treatments^58–60^. The extremely up-regulated expression of *GST* indicated that tissues and cells might have to face stress under light condition during shoot and regeneration of the callus. *ATGs* encode autophagy-related proteins which are required for autophagy process, and cooperate with jasmonate- and WRKY33-mediated signaling pathways in the regulation of plant defense responses to biotic and abiotic stresses^61^. Autophagy-defective mutant *atg2–2* exhibited powdery mildew resistance and mildew-induced cell death in *Arabidopsis*^62^. In apple, overexpression of *ATG18a* could promote drought tolerance through activating autophagy and reducing oxidation damage^63^. Thus, up-regulated expression of *GST* and *ATG18b* reflected the stresses and defense responses took place during redifferentiation of tea plant tissue, which might be modulated by *miR157d-5p* and *miR393c-3p*.

These miRNA targeted genes were mainly involved in transcription regulation and signaling transduction. Expression change of these genes would cascadedly influence the expression of downstream genes associated with cell division, proliferation and specialization, consequently alter the metabolism pathway related to the dedifferentiation and redifferentiation, such as the genes associated with “aminoacyl-tRNA biosynthesis”, “proteasome” and “glutathione metabolism”, “porphyrin and chlorophyll metabolism”, “mRNA surveillance pathway”, “nucleotide excision repair”, “protein export”, “2-oxocarboxylic acid metabolism” and “pyruvate metabolism”, finally lead to phase transition in tissue culture of stem explant.

## Conclusions

One hundred and ninety eight miRNAs were detected from the stem explant and its tissue culture derivatives, of which 178 miRNAs exhibited differential expression in various samples, 57 miRNAs exhibited phase-specific expression patterns. Expression of 12 phase-specific miRNAs and interaction of 6 miRNA/target gene modules were validated by qPCR. The *miR167d, miR156, miR396, miR166a-3p, miR157d-5p* and *miR393c-3p* might involve in regulation of dedifferentiation and redifferentiation of stem explant by targeting the *ERF003*, S*PB1, AIP15A, ATHB15, GST* and *ATG18b*, respectively.

## Methods and Materials

### Preparation of tea plant samples

Tissue culture seedlings of *C. sinensis* cultivar ‘Jinxuan’ were micropropagated and maintained on the Murashige and Skoog (MS) medium with addition of 2 mg/L 6-benzylaminopurine (BAP), 0.1 mg/L naphthalene acetic acid (NAA), 30 g/L sucrose and 9 g/L agar through single-node culture. The stem explant (S_Explant) were inoculated onto the callus inducing medium (CIM) for obtaining the callus (designated as S_Primary callus), and the stem-derived callus was then inoculated onto the root inducing medium (RIM) in order to regenerate the roots (designated as S_Root). The detailed culture operations were described as previously paper^37^. Meanwhile, the stem-derived callus was also inoculated onto the shoot inducing medium (SIM) in order to obtain the regenerated shoot. The SIM was prepared and autoclaved at 121 °C for 20 min after adding 3 mg/L BAP, 0.1 mg/L NAA, 30 g/L sucrose and 9 g/L agar into MS medium. The shoots were regenerated after inoculation of the stem-derived callus on the SIM for around 60 days. Sampling was then carried out and designated as S_Shoot. The obtained samples were immediately frozen in liquid nitrogen and stored at −80 °C for further use. All the tests were conducted triply.

### Total RNA extraction

Total RNAs for sequencing mRNAs and miRNAs were extracted from the S-Explant, S-Primary callus, S-Root and S-Shoot samples according to the manufacturer’s protocols of the RNAprep Pure Plant Kit (TIANGEN Biotech Co., Ltd., Beijing, China) and RNAiso Plus (TAKARA Bio Inc., Shiga, Japan), respectively. The purity and concentration of RNAs were checked by NanoPhotometer spectrophotometer (Implen, CA, USA) and Qubit 2.0 Flurometer (Life Technologies, CA, USA), respectively. Bioanalyzer 2100 system (Agilent Technologies, CA, USA) was also used for assessing the RNA integrity *via* RNA Nano6000 Assay Kit.

### Transcriptome analysis

The sequencing libraries were constructed by using 3 μg total RNAs for each sample according to the protocol of NEBNext Ultra RNA Library Prep Kit. The libraries were then sequenced on Illumina Hiseq 2500 platform. After removing low quality reads and the reads containing adapter and ploy-N from raw data, clean data were used to assemble transcripts through Trinity software^64^, and then function of assembled transcripts were annotated using BLAST software^65^ after comparing with the database of the NCBI nonredundant protein sequences (Nr), Protein family (Pfam), Eukaryotic Orthologous Group/Clusters of Orthologous Groups of proteins (KOG/COG), a manually annotated and reviewed protein sequences (Swiss-Prot), Kyoto Encyclopedia of Genes and Genomes (KEGG) and Gene Ontology (GO). FPKM values were calculated according to number of reads per 1,000 base lengths of a gene in a million reads and used to represent expression level of unigenes, and DEGseq R package (v 2.1.0) was used to measure the expression difference of two samples and the significant threshold was set as *q* value < 0.005 & |log2 (fold change)| > 1. Three biological replicates at each culture stage were used for transcriptome analysis.

### Small RNA library preparation and sequencing

After qualification of the RNAs, 1.5 μg RNAs of each sample were used to construct the sequencing library with a NEBNext Ultra Directional RNA Library Prep Kit for Illumina (NEB, USA) according to the manufacturer’s recommendations. In brief, sRNAs were firstly ligated with 3′ and 5′RNA adapters by using T4 RNA Ligase 1 & 2 (truncated) (Takara, Dalian, China). Ligated products were then transcribed into cDNAs by using a SuperScript II RT Kit (Invitrogen, USA). PCR amplifications were performed with primers that annealed to the ends of the adapters. Size selection of the PCR products were performed on PAGE gel, and sRNA libraries were recovered by gel-cutting and purified through AMPure XP Kit (Beckman Coulter, Australia). Finally, quality of the cDNAs libraries was ensured by examining the size, purity, and concentration through an Agilent2100 Bioanalyzer (Agilent Technologies, Santa Clara, CA, USA). The quality-checked libraries were then sequenced using a HiSeq X-Ten System (Illumina, SanDiego, CA, USA) in the Biomarker Technology Co. (Beijing, China, http://www.biomarker.com.cn). Three biological replicates at each culture stage were used for sRNA sequencing.

### Small RNA assembly and analysis

The quality of original data was verified after sequencing, and clean reads were obtained by removing the low-quality reads, reads with ploy-N content greater than or equal to 10%, and sequences shorter than 18nt or longer than 30nt and cutting off the 3′ joint sequence. In order to obtain unannotated reads containing miRNAs, through BowTie software^66^, the clean reads were aligned with Silva database, GtRNAdb database, Rfam database and Repbase database respectively to filter the ncRNAs including rRNA, tRNA, snRNA, snoRNA and repetitive sequences. The unannotated reads were then mapped with the reference transcriptome library to obtain positional information by BowTie software^66^. The amounts of common sequences and the type of unique sequences were counted between different samples.

TPM value was used as expression level of miRNA, which was calculated according to equation: TPM = read count *1000000/mapped reads. DEGseq R package was used to measure the miRNA expression difference between two samples. The differentially expressed miRNAs were obtained according to the threshold of *q* value < 0.005 & |log2(Fold change) > 1| between different samples.

### Identification of conserved and novel miRNAs

The mapped sequences were further used to align with the miRNAs from all species in the miRBase to identify the known miRNAs. miRDeep2^34^ was used to identify novel miRNAs from each sample, and to carry out the structural prediction of miRNAs and their precursors.

### Annotation of the miRNA targeted genes

Based on known and novel miRNAs as well as gene sequence information of corresponding transcriptome library, targeted genes were predicted by using TargetFinder software^67^. The predicted target genes were then compared with Nr, Swiss-Prot, GO, COG, KEGG, KOG and Pfam databases to obtain annotation information through BLAST software.

### Verification of the miRNAs and their targeted genes by qPCR

Expression of the mature known and novel miRNAs was verified through poly (A) RT-qPCR according to the method described by Shi and his colleagues^68^. qPCRs were carried out by using TB Green Premix Ex Taq II (Clontech, USA) on an Applied Biosystems StepOnePlus Real-Time PCR System (ABI, Carlsbad, CA, USA) according to the kit protocol. The reaction was performed at 95 °C for 30 s, and 40 cycles of 95 °C for 5 s and 60 °C for 30 s. The threshold cycle (Ct) was determined as the cycle number at which the fluorescence intensity passed a pre-determined threshold. Three biological samples for each treatment were used and all reactions were assayed in triplicate for each biological sample, and 5.8 S rRNA was selected as the reference gene^69,70^ after comparison had been conducted in our pre-experiment where expression of 5.8 S rRNA was much steadier and easier to be detected than U6 in each culture stage of tea plant. The primers for the miRNA qRT-PCR were shown in Supplementary Table S3.

To validate the expression of the miRNA targeted genes, qRT-PCR was also performed on Applied Biosystems StepOnePlus Real-Time PCR System by using SYBR Premix Ex Taq II Master Mix (TaKaRa Biotechnology Co., Ltd., Dalian, China) according to the manufacturer’s protocol. β-*Actin*^37^ was used as reference gene and relative expression of the targets was calculated using equation 2^−ΔΔct^. Detailed information about the primers used in this study was presented in Supplementary Table S4.

The data were statistically analyzed with SAS Version 9.0 software (SAS Institute, Cary, NC, USA) using Duncan’s multiple range test at significance level of p < 0.05.

## Supplementary information

Supplementary Information.

Supplementary Information 2.

SupplementaryInformation 3.

Supplementary Information 4.

Supplementary Information 5.

Supplementary Information 6.

## Data Availability

The RNA-Seq reads and assembling data had been submitted onto the GenBank (https://www.ncbi.nlm.nih.gov/Traces/study/?acc=PRJNA563232&o=acc_s%3Aa; https://www.ncbi.nlm.nih.gov/nuccore/GHXM00000000%20). Other datasets generated during and/or analyzed during the current study are available from the corresponding author on reasonable request.
