# Supplementary material for: MicroRNAs and their targeted genes associated with phase changes of stem explants during tissue culture of tea plant
Source: Sci Rep. 2019 Dec 27;9:20239. doi: 10.1038/s41598-019-56686-3 (PMC6934718; doi:10.1038/s41598-019-56686-3)

**Supplementary**

**Fig. S1.** GO analysis result of the targeted genes during phase change of tissue culture. A. S_Explant *vs* S_Primary callus; B. S_Root *vs* S_Primary callus; C. S_Primary callus *vs* S_Shoot.


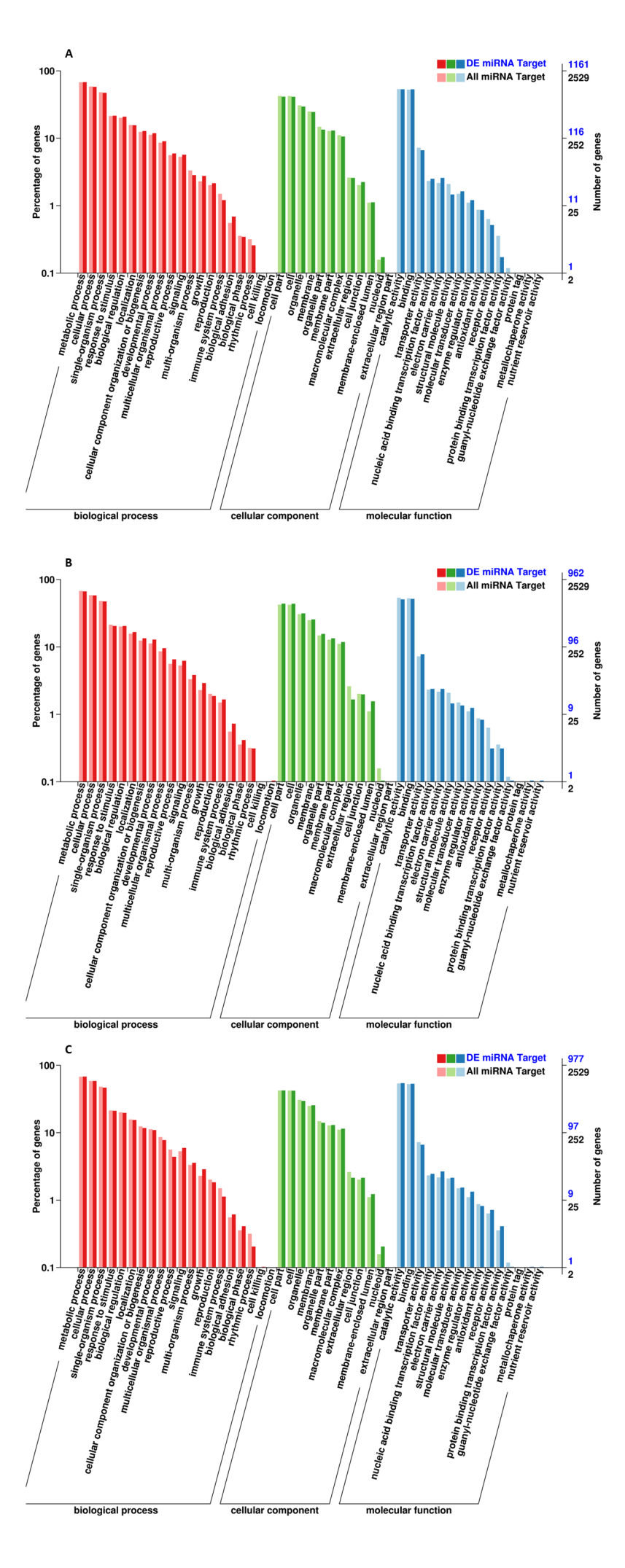

Supplement: Supplementary file 1 — Supplementary Information. [file 41598_2019_56686_MOESM1_ESM.docx]
