# Supplementary material for: MicroRNAs and their targeted genes associated with phase changes of stem explants during tissue culture of tea plant
Source: Sci Rep. 2019 Dec 27;9:20239. doi: 10.1038/s41598-019-56686-3 (PMC6934718; doi:10.1038/s41598-019-56686-3)

**Supplementary**

**Fig. S2.** The heat-map of 57 differentially expressed phase-special miRNA shared in the 4 samples, based on Z-score normalized TPM values in eight internode segments.


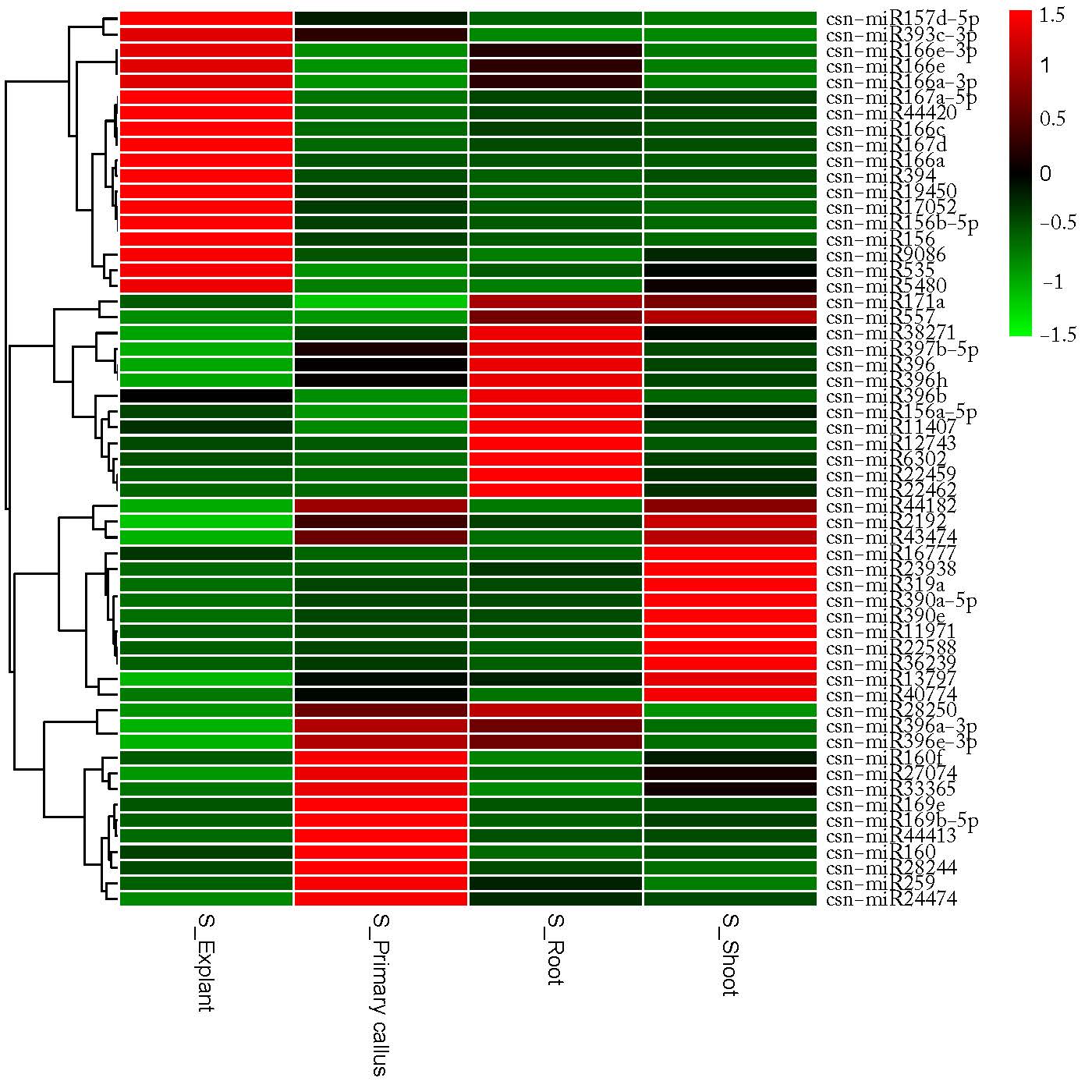

Supplement: Supplementary file 2 — Supplementary Information 2. [file 41598_2019_56686_MOESM2_ESM.docx]
