# Supplementary material for: MicroRNAs and their targeted genes associated with phase changes of stem explants during tissue culture of tea plant
Source: Sci Rep. 2019 Dec 27;9:20239. doi: 10.1038/s41598-019-56686-3 (PMC6934718; doi:10.1038/s41598-019-56686-3)
Supplement: Supplementary file 5 — Supplementary Information 5. [file 41598_2019_56686_MOESM5_ESM.docx]

**Table S3.** Information of primer for miRNA qRT-PCR.

| miRNA | qRT-PCR primer (5’-3’) | Length | TM |
| --- | --- | --- | --- |
| csn-miR156 | TTGACAGAAGATAGAGAGC | 19 | 47.6 |
| csn-miR156b-5p | TTGACAGAAGATAGAGAGCAC | 21 | 51.0 |
| csn-miR156a-5p | TGACAGAAGAGAGTGAGCAC | 20 | 54.0 |
| csn-miR157d-5p | TGACAGAAGATAGAGAGCAC | 20 | 50.6 |
| csn-miR160 | GCCTGGCTCCCTGTATGCCAT | 21 | 63.1 |
| csn-miR166a | TCGGACCAGGCTTCATTCCTC | 21 | 59.7 |
| csn-miR166a-3p | TCGGACCAGGCTTCATTCCCC | 21 | 62.7 |
| csn-miR166c | TCGGACCAGGCTTCATTCCT | 20 | 58.8 |
| csn-miR160f | TGCCTGGCTCCCTGTATGCC | 20 | 63.5 |
| csn-miR166e | GGACCAGGCTTCATTCCCC | 19 | 59.2 |
| csn-miR166e-3p | CTCGGACCAGGCTTCATTCCC | 21 | 61.2 |
| csn-miR167a-5p | TGAAGCTGCCAGCATGATCTA | 21 | 56.2 |
| csn-miR167d | TGAAGCTGCCAGCATGATCT | 20 | 56.9 |
| csn-miR169b-5p | CAGCCAAGGATGACTTGCCGG | 21 | 62.1 |
| csn-miR169e | AGCCAAGGATGACTTGCCGG | 20 | 61.1 |
| csn-miR171a | TGATTGAGCCGTGCCAATATC | 21 | 55.3 |
| csn-miR319a | CTTGGACTGAAGGGAGCTCC | 20 | 58.2 |
| csn-miR390a-5p | AAGCTCAGGAGGGATAGCGCC | 21 | 62.5 |
| csn-miR390e | AGCTCAGGAGGGATAGCGCC | 20 | 62.6 |
| csn-miR393c-3p | ATCATGCTATCCCTTTGGATT | 21 | 50.7 |
| csn-miR394 | TTGGCATTCTGTCCACCTCC | 20 | 57.8 |
| csn-miR396 | TTCCACAGCTTTCTTGAACTG | 21 | 52.7 |
| csn-miR396a-3p | GTTCAATAAAGCTGTGGGAA | 20 | 49.8 |
| csn-miR396b | TTCCACAGCTTTCTTGAACT | 20 | 51.2 |
| csn-miR396e-3p | GTTCAATAAAGCTGTGGGAAA | 21 | 50.2 |
| csn-miR396h | TCCACAGCTTTCTTGAACTG | 20 | 52.4 |
| csn-miR397b-5p | ATTGAGTGCAGCGTTGATGAA | 21 | 54.7 |
| csn-miR535 | TGACAACGAGAGAGAGCACGC | 21 | 60.0 |
| csn-miR_c259 | ATGGACTCTTCTAACTGTGAGAAG | 24 | 53.4 |
| csn-miR_c557 | GTGCTGTCTATCGTCGTCATG | 21 | 55.7 |
| csn-miR_c2192 | TTGCATACGCACCTGAATCGG | 21 | 58.2 |
| csn-miR_c5480 | ACTTTCCGGCCTGTCTTCGGC | 21 | 63.4 |
| csn-miR_c6302 | GTTCAAGGAAGCTGTGGGAAG | 21 | 56.3 |
| csn-miR_c9086 | TGGATGTAGCAAAGAGAAGCT | 21 | 53.3 |
| csn-miR_c11407 | TTTCCAAGTCCACCCATTCCTA | 22 | 55.6 |
| csn-miR_c11971 | TCCGATCATTAATCGTCGCAGATC | 24 | 56.6 |
| csn-miR_c12743 | GAATGTTGGATCCTTTGAGGTC | 22 | 53.3 |
| csn-miR_c13797 | CTTCCATACGTCAGGAGCTGC | 21 | 58.4 |
| csn-miR_c16777 | GTGCTCTCTATCGTCGTCATG | 21 | 55.2 |
| csn-miR_c17052 | TCCCTTTGGATGTCGTCCTGT | 21 | 58.5 |
| csn-miR_c19450 | TGACTCTTGGGTTGTTGAGGC | 21 | 57.7 |
| csn-miR_c22459 | TGAATGGTATGAATCACTTTA | 21 | 45.0 |
| csn-miR_c22462 | TGAATGGTATGAATCACTTTG | 21 | 47.0 |
| csn-miR_c22588 | CTATTATCAAGATTCTGTGGTG | 22 | 47.5 |
| csn-miR_c23938 | CTGTGTTTGGATCATGGATTT | 21 | 50.1 |
| csn-miR_c24474 | GGTTAACATTGTTGCTAACTGTGG | 24 | 54.1 |
| csn-miR_c27074 | TAATTTACTGAGAATTTATTGAGT | 24 | 43.7 |
| csn-miR_c28244 | TTGTGTTTTATTTCTGTGCAAGTA | 24 | 49.8 |
| csn-miR_c28250 | ATGGAAGCAACAAAACAACCTCTGC | 25 | 58.4 |
| csn-miR_c33365 | TTTTTGCTTTCTGTTTTCATT | 21 | 45.2 |
| csn-miR_c36239 | CGGGTGTACTGGGCAACC | 18 | 59.8 |
| csn-miR_c38271 | ATTTTTGAATGAAAGGCCCATGTG | 24 | 54.0 |
| csn-miR_c40774 | GCAACTTATGACAAGGAGGTAGAG | 24 | 54.9 |
| csn-miR_c43474 | GTTAATAGGACTGATTGGTAAGTA | 24 | 48.5 |
| csn-miR_c44182 | ACAAAAATGATTGTTCAACTAAC | 23 | 46.3 |
| csn-miR_c44413 | ATTGAACTCTGTTATCAAGATTAAC | 25 | 47.7 |
| csn-miR_c44420 | GTGCTCTCTCTCGTTGTCATA | 21 | 53.8 |
| 5.8SrRNA | ACGTCTGCCTGGGTGTCACAA | 21 | 62.0 |
