# Supplementary material for: MicroRNAs and their targeted genes associated with phase changes of stem explants during tissue culture of tea plant
Source: Sci Rep. 2019 Dec 27;9:20239. doi: 10.1038/s41598-019-56686-3 (PMC6934718; doi:10.1038/s41598-019-56686-3)
Supplement: Supplementary file 6 — Supplementary Information 6. [file 41598_2019_56686_MOESM6_ESM.docx]

**Table S2.** Information of primers for mRNA qRT-PCR.

| Gene | Annotation | Primer pair |
| --- | --- | --- |
| *ERF3* | Ethylene-responsive transcription factor ERF003 | ERF003F 5’-GCAGCTTCATCGTACGCTCT  ERF003R 5’-CTCCTCCAAGACCGCTTCAC |
| *SBP1* | Squamosa promoter-binding protein 1 | SBP1F 5’-GCTAAGCTTGCAGTTGCTGT  SBP1R 5’-ATCAAACGCCTGCGACAACT |
| *ATHB15* | Homeobox-leucine zipper protein ATHB-15 | ATHB-15F 5’-TGGTCTTCGGCTTCCTTTGG  ATHB-15R 5’-CTGAGTGGTGCCAGAGTGTT |
| *AIP15A* | Auxin induced protein 15A | AIP 15A F 5’-TCATGGCTGACCCTTCTCTT  AIP 15A R 5’-CTCAGCAGGCTCTTGTCTCC |
| *ATG18b* | Autophagy-related protein 18b | ATG 18bF 5’-AGAAAGAACCATCGCAGCCA  ATG 18bR 5’-TTTTGGCTCTTCCAGCGAGT |
| *GST* | Glutathione S-transferase | GSTsF 5’-TCCTGTTTGGGAGGAAGTTGG |
|  |  | GSTsR 5’-TTTTCTGGCAGCAGCAATGAG |
